# Supplementary material for: Prioritizing COVID-19 vaccination efforts and dose allocation within Madagascar
Source: BMC Public Health. 2022 Apr 12;22:724. doi: 10.1186/s12889-022-13150-8 (PMC9002044; doi:10.1186/s12889-022-13150-8)

Figure S1: Modeling framework. The core of the model is similar to that of Roche et al. 2020. The same model has been applied to understanding the covid dynamics and the effectiveness of NPI at a national level in Madagascar (Evans et al. 2020). The stochastic simulation includes eight states: Susceptible (S), Exposed (E), Infected (symptomatic, I), Infected (Asymptomatic, A), and recovered from asymptomatic (U),recovered from symptomatic (R) infected symptomatic individuals who become severely ill (M) and infected symptomatic individuals who die (D) [17]. The population is divided into seven age classes (0-9;10-19;20-29;30-39;40-49;50-59; 60+) . Susceptible individuals in an age class i (S_i) can get the infection according to the basal transmission rate ($\boldsymbol{\beta}$), the number of infectious (symptomatic and asymptomatic) individuals in each age class, and the contact rate among age classes. Once infected, an individual becomes infectious after an incubation period of 3 days, i.e. at a rate ε = 1/3 days. Infectious individuals can be symptomatic (I) with a probability of *p* (assumed to be 40%) or asymptomatic (A) otherwise. Infectious individuals (I and A) can recover at a rate σ = 1/5 days. Infectious symptomatic have a probability α to become severely ill and a probability (π_i) dependent on their age class to die from the disease (D).

We included vaccination into the model. The daily number of people vaccinated (*n*) depends on the number of healthcare workers and the total number of individual vaccinated is limited by the acceptance (η_i). Individuals in the compartment S, E, A, U, and R can be vaccinated. However, vaccinating non-susceptible individuals would lead to vaccine wastage. Thus, we only track the number of susceptibles n_S that are vaccinated. Without testing, n_S is drawn from a binomial distribution Binomial(n, S/(S + E + A + U +R). We consider two scenarios to test for seroprevalence and thus avoid vaccine wastage (see main text). Additionally, vaccination can prioritize older individuals or distribute the doses randomly in each age class according to a multinomial distribution where the vector of probabilities are given by the frequency of the individuals in each age-class. Vaccinated individuals gain protection after a lag of 6-days

according to the vaccine efficacy (ν) which we assumed to be at 76% to approximate the clinical vaccine efficacy against symptomatic infection seen for the ChAdOx1 nCoV-19 (AZD1222). The actual number of people gaining protection is Binomial(n_S,ν) .

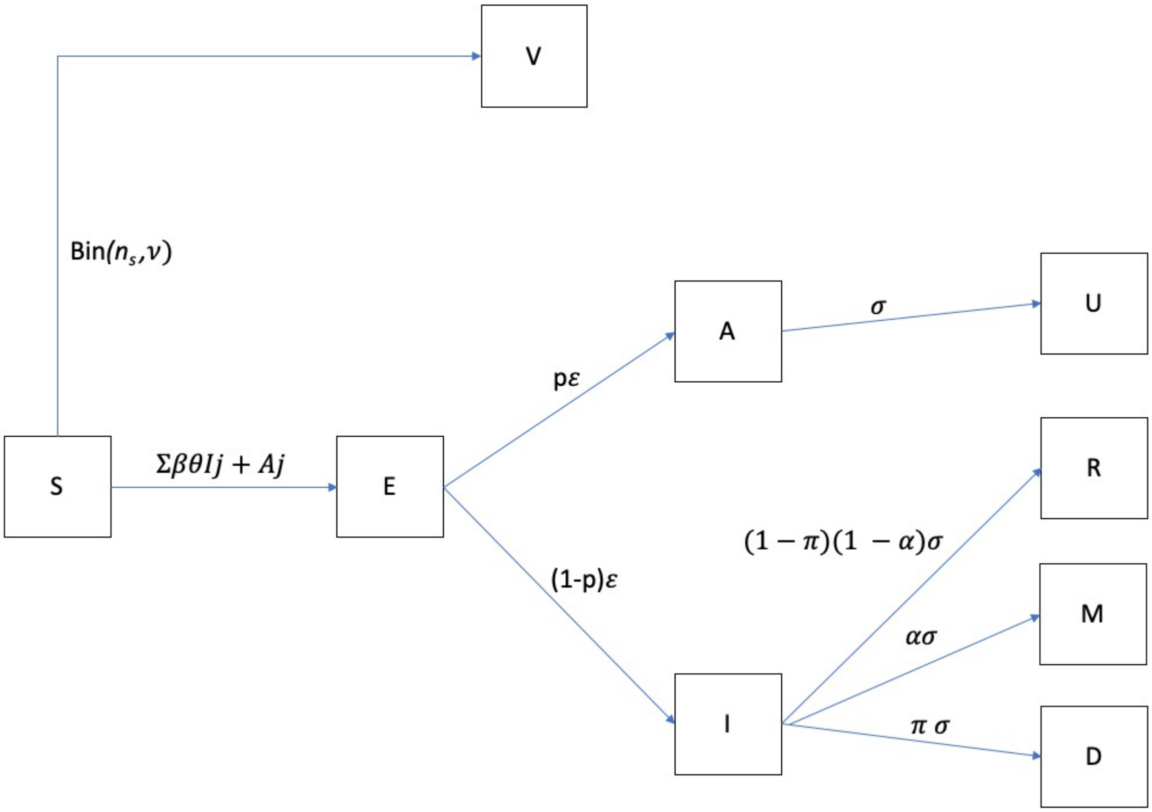

Supplement: Supplementary file 1 — Additional file 1: Figure S1. Modeling framework. [file 12889_2022_13150_MOESM1_ESM.pdf]
