# Supplementary material for: Prioritizing COVID-19 vaccination efforts and dose allocation within Madagascar
Source: BMC Public Health. 2022 Apr 12;22:724. doi: 10.1186/s12889-022-13150-8 (PMC9002044; doi:10.1186/s12889-022-13150-8)

Figure S2: **The estimated reduction in mortality for each vaccine allocation strategy given different R0.** The reduction in mortality by allocation strategy for stochastic simulations assuming vaccine acceptance of 70%, rollout speed where 50% of health care workers were mandated to vaccinate 20 people a day, start day of 10 days following initial seeding event, and the number of total doses equals 20% of the population with A) R0=2.5 as estimated in [16] and B) R0=5.08 as estimated for the Delta variant [19].

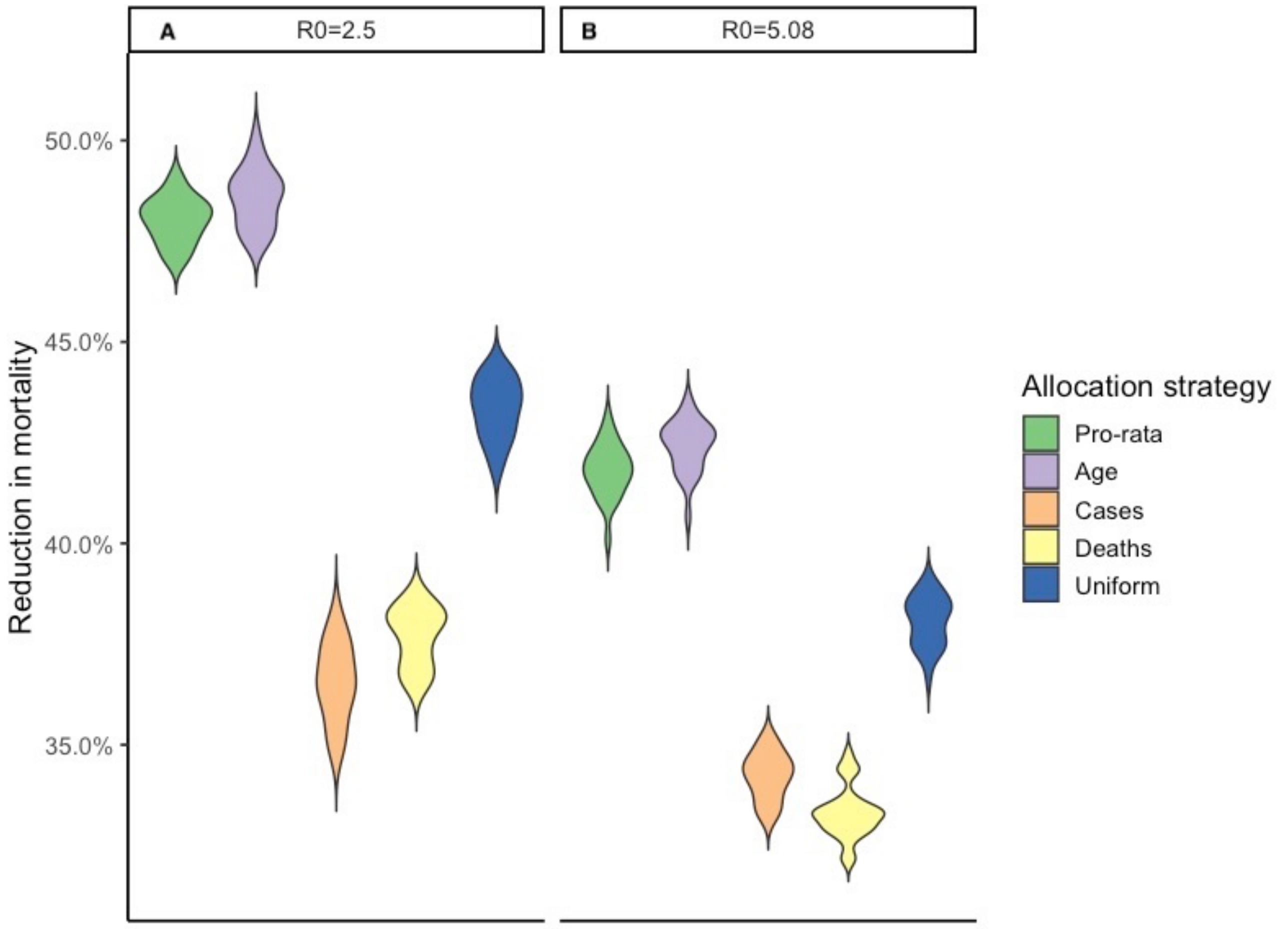

Supplement: Supplementary file 2 — Additional file 2: Figure S2. The estimated reduction in mortality for each vaccine allocation strategy given different R0. [file 12889_2022_13150_MOESM2_ESM.pdf]
